# Suppressing Interface Defects in Perovskite Solar Cells via Introducing a Plant-Derived Ergothioneine Self-Assembled Monolayer

**DOI:** 10.3390/ma17235739

**Published:** 2024-11-23

**Authors:** Cheng-Hsien Yeh, Hung-Chieh Hsu, Jung-Che Tsao, Hsuan-Ta Wu, Teh-Pei Lin, Chien-Te Wu, Shih-Hsiung Wu, Chuan-Feng Shih

**Affiliations:** 1 Department of Electrical Engineering, National Cheng Kung University, Tainan 70101, Taiwan; n28084012@mail.ncku.edu.tw (C.-H.Y.); n28084020@gs.ncku.edu.tw (H.-C.H.); jerry881015@gmail.com (J.-C.T.); 2 Applied High Entropy Technology (AHET) Center, National Cheng Kung University, Tainan 70101, Taiwan; 3 Green Energy and Environment Research Laboratories, Industrial Technology Research Institute, Tainan 711010, Taiwan; 4 Department and Institute of Electrical Engineering, Minghsin University of Science and Technology, Hsinchu 30401, Taiwan; htwu@must.edu.tw; 5 Institute of Green Products, Feng Chia University, Taichung 40724, Taiwan; drtplin@gmail.com (T.-P.L.); ctwu168@gmail.com (C.-T.W.); 6 Hierarchical Green-Energy Materials (Hi-GEM) Research Center, National Cheng Kung University, Tainan 70101, Taiwan

**Keywords:** interface, perovskite, L-Ergothioneine, passivation, aging test, long-term stability

## Abstract

Perovskite solar cells are among the most promising renewable energy devices, and enhancing their stability is crucial for commercialization. This research presents the use of L-Ergothioneine (L-EGT) as a passivation material in perovskite solar cells, strategically placed between the electron transport layer and the perovskite absorber layer to mitigate defect states at the heterojunction interface. Surface analysis reveals that introducing L-EGT passivation material significantly improves the quality of the perovskite film. X-ray diffraction analysis indicates that L-EGT slows down perovskite film degradation and successfully suppresses secondary phase formation. X-ray photoelectron spectroscopic analysis shows that oxygen vacancies in the lattice decrease from 29.21% to 15.81%, while Ti^4+^ content increases from 70.75% to 79.15%, suggesting that L-EGT effectively passivates trap states at the interface between perovskite and TiO_2_ electron transport layer. The reduction of defects at the interface inhibits charge accumulation and lowers the device’s internal series resistance, leading to improved overall performance. The study finds that the introduction of L-EGT significantly improves the fill factor and efficiency, with the power conversion efficiency (PCE) rising from 16.88% to 17.84%. After 720 h of aging, the PCE retains approximately 91%. The results demonstrate the significant impact of the amino acid L-EGT passivation material in suppressing interfacial defects and greatly improving the long-term stability of perovskite devices.

## 1. Introduction

Perovskite solar cells have garnered significant research interest due to their unique photovoltaic properties, such as tunable bandgaps, high absorption coefficients, long carrier lifetimes, and long carrier diffusion lengths, combined with their low carbon footprint [1,2,3,4]. Currently, the highest efficiency achieved by perovskite solar cells stands at 26.7%, nearing that of traditional silicon-based solar cells. However, the inherent disadvantages of organic perovskite materials make them susceptible to degradation caused by environmental factors such as humidity, light exposure, and temperature fluctuations. This degradation not only compromises the quality of the perovskite layer but also elevates defect states at the interface between the transport and perovskite layers, both of which represent major bottlenecks in improving the efficiency and long-term stability of perovskite photovoltaic devices. In response to these challenges, recent efforts have focused on developing passivation materials to be introduced at the junction between the electron/hole transport layers and the absorber layer. These materials aim to suppress interfacial defect states, enhance interfacial interactions, slow down the degradation of the perovskite layer, refine energy-level alignment between perovskite and electron transport layers, diminish series resistance within the photovoltaic device, and minimize the probability of carrier radiative recombination. Amino acid-based materials have been reported as effective in passivating interfacial defects, thereby enhancing perovskite crystallinity, promoting nucleation, and forming dense perovskite films [5,6,7,8]. For instance, J. Du et al. introduced glycine as an interfacial buffer layer, successfully improving the characteristics of the heterojunction between the electron transport layer and the perovskite, leading to enhanced device stability [5]. M. Wu et al. proposed a multifunctional small-molecule interfacial passivator to modify the interface (electron transport layer/perovskite), investigating its effects on carrier transport, perovskite growth, defect passivation, and solar cell performance [9]. Additionally, F. Han et al. utilized bifunctional 4-pyridinecarboxylic acid (4-PA) to modify the interfacial quality. The COOH group in 4-PA established chemical bonds with TiO_2_, enhancing the extraction ability of the electron transport layer, reducing charge accumulation at the interface of perovskite film, and minimizing hysteresis of the device, which resulted in escalating PCE from 14.65% to 18.9% [10]. These studies underscore the critical role of passivation materials in mitigating defects between the perovskite and the electron transport layer.

It is found that the anchoring group (–COOH) of the self-assembled monolayer (SAM) molecules interacts with the metal oxide TiO_2_, forming a chemical bond with the substrate surface, which is crucial in this process. The anchoring mechanism demonstrates varying binding energies, which are essential for interfacial charge transfer properties and overall device performance. L-EGT, a plant-derived amino acid compound, was chosen based on previous studies indicating that organic molecules with multiple functional groups can effectively passivate defects [11,12,13,14]. Ergothioneine molecules interact strongly with the perovskite lattice, leading to improved film quality and reduced defect density [15]. It was found that the −NH_2_ groups provided by amino acid materials form hydrogen bonds with the MA ions in the perovskite composition, while the carboxyl (–COOH) groups mitigate to diminish oxygen vacancies within TiO_2_. Furthermore, hydrogen bonding has been shown to effectively reduce defects by stabilizing halide ions [16]. The ionic nature of the perovskite lattice facilitates molecular coordination with defect sites via Lewis acid-base chemistry, thereby passivating interfacial defects [17,18,19,20]. The Lewis groups in L-EGT interact strongly with uncoordinated Pb^2+^ ions in the perovskite, which are known to induce non-radiative carrier recombination and negatively affect perovskite stability. Passivating uncoordinated Pb^2+^ defects enhances both the stability and electrical performance of the perovskite [21,22,23]. Furthermore, our findings demonstrate competitive stability when compared with similar perovskite solar cell structures. M. Li et al. used dipeptide molecules rich in functional groups as defect passivation agents for perovskite films, achieving retention of approximately 75–77% of the initial efficiency in MAPbI_3_ perovskite solar cells after 720 h [24]. Additionally, we compared our results with other studies using MA-based perovskites with the same n-i-p device structure. For instance, A. Kumar et al. employed trichloroacetic acid (TCAA) in an anti-solvent mixture to fabricate low-defect MAPbI_3_ perovskite solar cells, which retained around 84% of initial efficiency after 750 h [25]. Similarly, J. Zhang et al. used dipole molecules to modify the MA-based perovskite interface, maintaining about 89% of initial efficiency after 720 h [26].

This study introduces the self-assembled monolayer (SAM) of L-EGT as a passivation material positioned between the perovskite layer and the electron transport layer. The objective is to mitigate defects at the perovskite/TiO_2_ transport layer interface and boost the electrical performance of solar cells. Furthermore, a large-area blade-coating technique was employed to fabricate perovskite solar cells. The results indicated substantial enhancement in the overall power conversion efficiency, open-circuit voltage (V_oc_), and fill factor (FF) of perovskite devices following the introduction of L-EGT. After 720 h of aging tests, the remaining PCE of the device also retained 91% of its initial value, indicating long-term stability.

## 2. Materials and Methods

### 2.1. Materials

Methylammonium iodide (MAI) was procured from GreatCell Solar, lead (II) iodide (PbI_2_, 99.9985%) from TCI (Tokyo, Japan), and Spiro-OMeTAD (>99.5%) from Derthon (Shenzhen, China), while dimethyl sulfoxide (DMSO, 99%) and gamma-butyrolactone (GBL) were supplied by JT Baker (Radnor, PA, USA). All materials were used as received without further purification. The organic perovskite precursor was prepared in a nitrogen-filled glove box, maintaining oxygen levels <1 ppm and humidity under 30%. The perovskite precursor was made with a 7:3 GBL and DMSO solvent mixture, stirred for uniformity, and filtered through a filter.

### 2.2. Device Fabrication

Firstly, a fluorine-doped tin oxide (FTO) glass substrate underwent etching with zinc powder and hydrochloric acid (aq), followed by a series of ultrasonic cleans in acetone, ethanol, and deionized water. After drying with dry air, a compact TiO_2_ layer was formed using a TiCl₄ treatment. Subsequently, a mesoporous TiO_2_ layer was applied over the compact layer using a doctor-blade technology. Subsequently, the TiO_2_-coated substrates were then annealed at 500 °C for one hour. L-EGT self-assembled monolayers were created through a soaking process; the chemical structure of L-Ergothioneine is shown in Figure S1a. The perovskite precursor, MAPbI_3_, was then applied to the electron transport layer, followed by the spin-coating of a Spiro-OMeTAD solution in a dry-air glove box (with relative humidity below 10%). Lastly, an Au electrode layer was deposited by thermal evaporation to finalize the perovskite device. The completeness structure of perovskite solar cells is presented in Figure S1b.

### 2.3. Characterizations

The microstructural characteristics of perovskite thin films were investigated using a scanning electron microscope (SEM, Hitachi SU8000, Tokyo, Japan), and surface profiling at the nanoscale was conducted using an atomic force microscope (AFM) (Bruker Dimension Icon, Karlsruhe, Germany). The crystallinity and phase configuration of the perovskite material were evaluated using X-ray diffraction (XRD, Bruker D8, Karlsruhe, Germany) with 0.02° angular resolution and utilizing Cu–Kα radiation. Transmittance spectra were recorded by UV–Vis spectrophotometers (HITACHI U-2800, Tokyo, Japan). The space-charge-limited current (SCLC) was analyzed through I–V measurements performed in dark conditions using a Keithley 2400 source meter (Washington, DC, USA). Current density–voltage (J–V) characteristics were recorded under AM 1.5G illumination at 100 mW/cm^2^ using an Enlitech SS-F5-3A solar simulator (Kaohsiung City, Taiwan), with the source meter Keithley 2400 (Washington, DC, USA) scanning from +1.1 V to −0.1 V (scan rate of 0.05 V/s). The steady-state photoluminescence (PL) and time-resolved photoluminescence (TR-PL) spectra were recorded using a Princeton Instruments Acton 2150 (Princeton Instruments, Acton, MA, USA) spectrophotometer to evaluate optical emission properties. X-ray photoelectron spectroscopy (XPS) analysis was performed using a PHI VersaProbe 4 with Al Kα radiation, and ^1^H NMR spectra were acquired on a Bruker AVANCE III HD 600 MHz NMR spectrometer. In the current–voltage measurements, each parameter is based on a sample size of at least 10 data points. For PL, TRPL, XRD, XPS, and ^1^H NMR analyses, each parameter is based on a sample size of at least 5 measurements to enhance data reliability. Each perovskite cell device has dimensions of 15 mm × 15 mm.

## 3. Results

**Figure 1:**
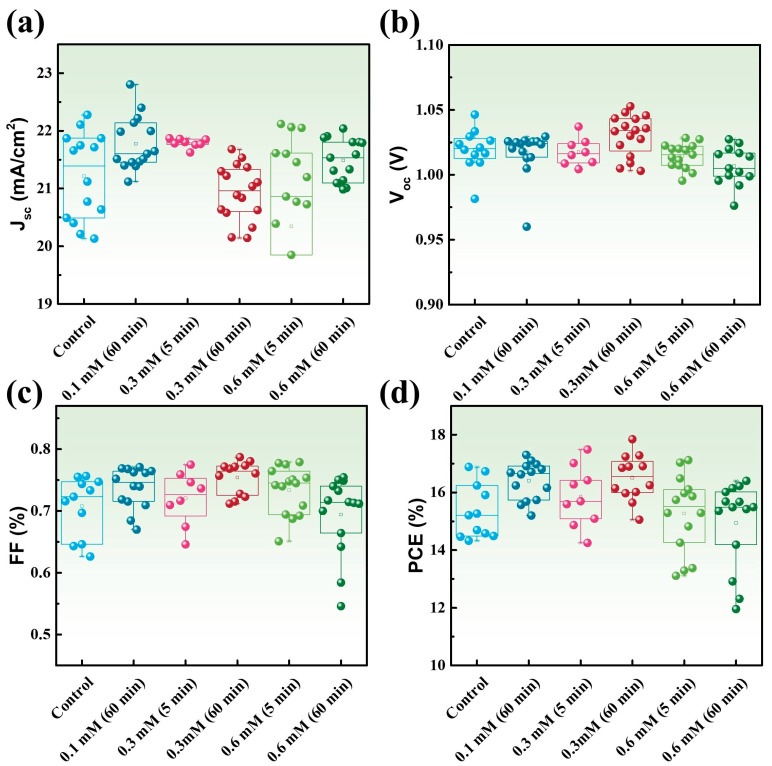
The (**a**) J_sc_ (**b**) V_oc_ (**c**) FF (**d**) PCE of the perovskite solar cell under varying concentrations and soaking durations of L-Ergothioneine.

First, Figure 1a–d present the effects of varying L-EGT soaking times and concentrations of L-EGT on device performance. The experimental outcome indicated that the optimal concentration of the L-EGT solution was determined to be 0.3 mM, with a soaking time of 60 min. From the boxplot trends, it was observed that both the PCE and the FF significantly improved as the concentration increased to 0.3 mM. The rise in the FF suggests that the addition of L-EGT enhanced the quality of interface, leading to decreased charge accumulation within the electronic states of the perovskite [27]. However, a decrease in J_sc_ was noted when the L-EGT concentration exceeded 0.3 mM. The L-EGT forms an ultrathin layer deposited on the TiO_2_ layer, creating a slim passivation monolayer at the perovskite/electron transport layer interface, which somewhat limits current characteristics [8]. Therefore, optimizing the deposition concentration and time for the self-assembled layer is crucial for the performance of the device. Based on the evaluation of the effects of L-EGT on V_oc_, FF, and PCE, the optimal parameters were a soaking concentration of 0.3 mM and a soaking time of 60 min.

**Figure 2:**
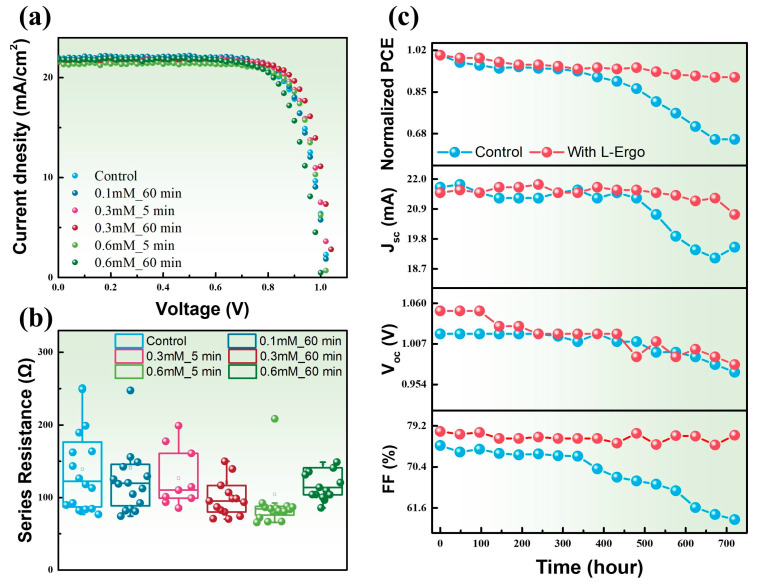
(**a**) The current–voltage curve of the perovskite solar cell under varying concentration and soaking duration of L-Ergothioneine. (**b**) The series resistance of perovskite solar cells under varying concentrations and soaking durations of L-Ergothioneine. (**c**) Long-term aging test of electrical properties of perovskite solar cells with and without (control) L-Ergothioneine.

Figure 2a presents the I–V curves for different L-EGT soaking times and concentrations of L-EGT. The data clearly indicate that the introduction of L-EGT led to significant improvements in both V_oc_ and FF. For perovskite solar cells treated with 0.3 mM L-EGT for 60 min, the V_oc_ increased from 1.02 V to 1.05 V, and the FF improved from 75% to 78%, resulting in an enhancement of the PCE from 16.88% to 17.84%. Table S1 presents the photovoltaic performance of perovskite solar cells subjected to various soaking durations and L-EGT concentrations. Figure 2b shows the series resistance (Rs) of perovskite solar cells fabricated under varying L-EGT soaking conditions. The series resistance is calculated from the current–voltage curve by taking the tangent at the open-circuit voltage (V_oc_) point; the reciprocal of the tangent slope at this point represents the series resistance. The reduction in Rs indicates improved carrier transport properties of the electron and hole transport layers, as well as enhanced contact between the transport layer and the conductive substrate. A lower Rs facilitates faster extraction of electrons and holes to the transport layers, thereby reducing radiative recombination and preventing the deterioration of the electrical performance of the device [28,29].

Figure 2c presents the stability trend of the perovskite device in ambient conditions after the introduction of L-EGT. The 720-hour aging test, as shown in Figure 2c, reveals the normalized trends of efficiency, J_sc_, V_oc_, and FF. The perovskite device without L-EGT retained only 65% of its initial PCE after 720 h of aging. Up to the first 400 h, there was no significant difference in aging characteristics between devices with and without L-EGT; however, beyond 400 h, the L-EGT-incorporated device maintained up to 95% of its initial PCE. In this research, the device incorporating L-EGT maintained 91% of its initial power conversion efficiency (PCE) even after 720 h of aging. From the FF aging trend, it was observed that the L-EGT-treated device retained the FF of approximately 80% after 720 h, whereas the FF of the untreated device significantly dropped as aging progressed, suggesting that interfacial properties deteriorated, likely due to perovskite degradation. Figure S2 analyzes the I–V characteristics of perovskite devices over various aging durations (0, 240, 480, 720 h). It was observed that the untreated device showed not only a notable decrease in V_oc_ and J_sc_ after 720 h but also a significant reduction in FF, indicating that increased interfacial defects between the perovskite and the transport layer might be responsible for these changes. Z. Qi et al. introduced L-EGT into carbon-based CsPbIBr_2_ perovskite solar cells, significantly improving the light-induced degradation resistance, with 70% of the initial PCE retained after 150 h of continuous illumination, indicating that L-EGT effectively inhibited photo-degradation in perovskite solar cells [15]. M. Hou et al. proposed the DA SAM-modified devices exhibit greater stability, making this interfacial engineering approach promising for developing stable PSCs, and the device retained 80% of its initial PCE after 300 h [30]. H. Kouki et al. employed amino-based self-assembled monolayers (SAMs) to modify the interfacial properties on the ZnO electron transport layer. Under 0.7 sun solar simulation, the device retained 67% of its initial efficiency after 148 h [31]. Similarly, C.-T. Lin et al. integrated a range of amino acid derivatives into MAPbI_3_-based perovskite solar cells to diminish photodegradation caused by oxygen exposure. As a result, they were able to preserve 78% PCE after 120 h of stability testing under ambient conditions [6]. J. Du et al. applied glycine as a buffer layer to improve the SnO_2_/CH_3_NH_3_PbI_3_ heterojunction, alleviating interfacial strain from lattice mismatch and suppressing crystalline defects, which resulted in 78% of the initial efficiency being retained after 840 h under ambient conditions (RH ≤ 30%) [5]. Compared to the results, the perovskite solar cell incorporating L-EGT retained up to 91% of their initial PCE after 720 h, with the FF remaining close to 80%, demonstrating the effectiveness of L-EGT in suppressing interfacial defects. The aging characteristics of various SAM materials have been comprehensively compiled in detail in Table S2.

**Figure 3:**
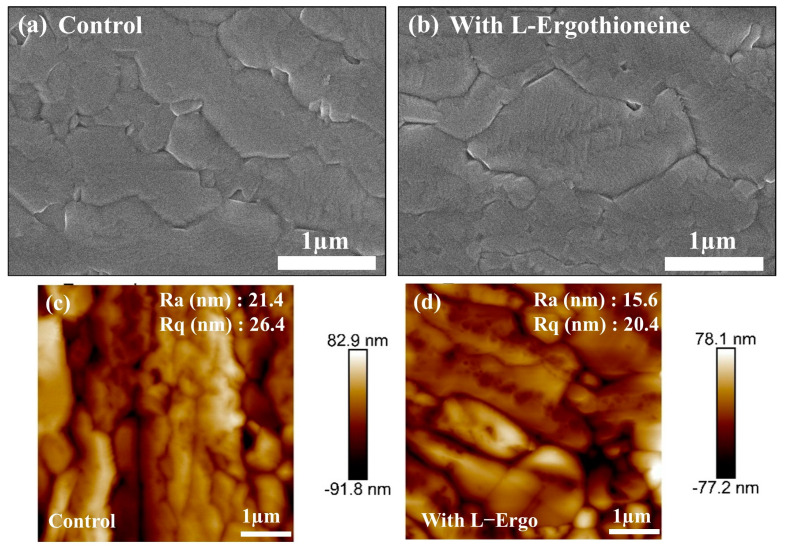
SEM top-view morphology images of perovskite films: (**a**) without L-Ergothioneine (control) and (**b**) with L-Ergothioneine. Atomic force microscopy images: (**c**) without L-Ergothioneine (control) (**d**) with L-Ergothioneine.

In this work, the L-EGT film was fabricated through a soaking technique. Figure 3a shows the SEM image of the perovskite morphology for the control sample without L-EGT, while Figure 3b presents the SEM image of the perovskite film coated with L-EGT at a concentration of 0.3 mM and a soaking time of 60 min. Compared to the control, the surface morphology of the perovskite film deposited on L-EGT showed a significant reduction in voids at the grain boundaries and a marked increase in grain size. This indicates that the introduction of L-EGT onto the TiO_2_ surface improved surface wettability, thereby enhancing grain boundary mobility, which promotes the formation of larger grains [32].

Figure 3c,d display surface roughness analyses, with Figure 3c representing the control and Figure 3d showing the surface roughness morphology of the perovskite layer deposited on L-EGT. After the incorporation of L-EGT, the surface roughness (RMS) of the perovskite thin film obviously reduced from 26.4 nm to 20.4 nm. In addition to altering the wettability of the substrate surface, the self-assembled monolayer (SAM) also influenced the growth process and quality of the perovskite film, resulting in improved surface uniformity. The surface roughness of the perovskite film influences the charge transfer efficiency and adhesion between the perovskite layer and the hole transport layer (Spiro-OMeTAD). Consequently, a reduction in surface roughness leads to better compatibility at the interface, which in turn improves charge carrier transfer properties and enhances the overall performance of the device [33].

**Figure 4:**
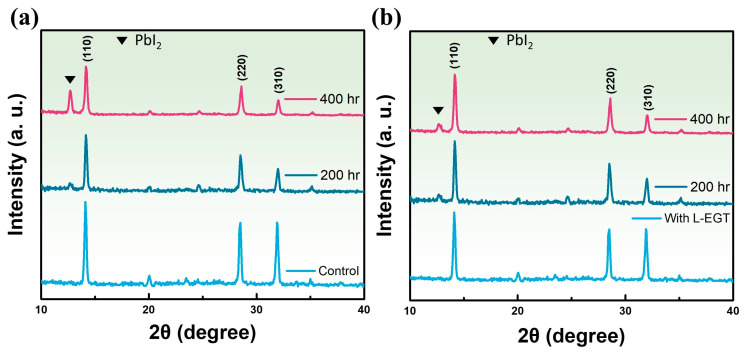
The X-ray diffraction pattern of perovskite thin film (**a**) without L-Ergothioneine (control) and (**b**) with L-Ergothioneine at different aging times.

Figure 4 presents the XRD diffraction patterns, where the crystallinity and compositional changes of the perovskite film following the introduction of L-EGT can be observed. The characteristic peaks of the perovskite are observed at the (110), (220), and (310) planes, and the symbol presents the signal of the PbI_2_ peak. Following the incorporation of L-EGT, the diffraction pattern showed no evidence of secondary phases, and the intensity of the (110), (220), and (310) peaks was consistent with that of structure without L-EGT, suggesting that the introduction of L-EGT did not change the structure of the perovskite. Figure 4a shows the XRD spectrum of a perovskite film without L-EGT, where a significant reduction in peak intensity was observed after 200 h of aging. After 400 h, the formation of a PbI_2_ secondary phase was detected at 12.8°. In Figure 4b, the perovskite film incorporating L-EGT showed only minimal intensity of PbI_2_ secondary phase formation after 400 h of aging testing. This observation indicates that the introduction of L-EGT effectively passivated the defects of uncoordinated Pb^2+^ within the perovskite film, prolonging its lifetime and suppressing PbI_2_ formation, which contributes to the improved aging characteristics of perovskite solar cells [34,35]. The electron cloud density distribution of different functional groups exhibits tunable characteristics related to interface passivation. The CO group, provided by carboxyl groups, is characterized by a high electron density, allowing CO to form a stronger Pb–O coordination with uncoordinated Pb^2+^ double bonds. This interaction helps reduce lead-based defects at the interface. Additionally, the strong electron-withdrawing ability of CO facilitates its role as nucleation centers, moderating the growth rate of the perovskite layer and promoting the formation of a high-quality perovskite absorber layer with larger grains [21,36,37]. The XRD reveal that the inclusion of L-EGT inhibited perovskite degradation and mitigated decline in FF for the perovskite devices.

**Figure 5:**
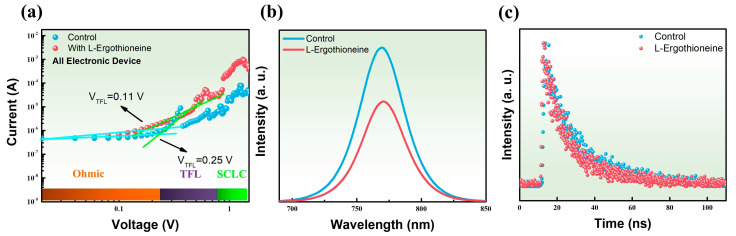
(**a**) Current density–voltage curves and trap density of perovskite films with and without L-Ergothioneine. (**b**) PL, (**c**) TRPL spectra of perovskites thin film deposited on TiO_2_ film with and without L-Ergothioneine modification.

Figure 5a presents the photocurrent characteristics recorded in dark conditions to evaluate the defect density and the trap-filled limit voltage (V_TFL_) of devices featuring electron-only structure. The graph displays three distinct regions: ohmic (I ∝ V), trap-filled limit (I ∝ Vn, where n > 2), and the space-charge-limited current (I ∝ V^2^). Notably, in the trap-filled region, there is an obvious nonlinear rise in current, signaling that all trap states have been occupied by injected carriers. The voltage at the boundary between the ohmic and trap-filled regions corresponds to the VTFL. The density of trap defects within the perovskite film is determined using the formula VTFL = (e × Nt × d^2^)/(2 × ε × ε₀), where e represents the electron charge, Nt the trap density, d the thickness of the perovskite film, ε the dielectric constant of the perovskite, and ε₀ the permittivity of free space [38]. Upon introducing L-EGT, VTFL decreased significantly from 0.25V to 0.11V. In the TiO_2_/L-EGT/MAPbI_3_ configuration, the VTFL was measured at 0.11V, and the trap density (Nt) was calculated to be 2.43 × 10^15^ cm^−3^, indicating a reduction in defect density within the electronic structure. The presence of various trap states at the interfaces and grain boundaries in perovskite solar cells contributes to non-radiative recombination, which diminishes both carrier lifetime and density, ultimately leading to degraded device performance. Therefore, incorporating a self-assembled monolayer (SAM) at the interface can passivate crystalline defects in the perovskite film. For instance, Lewis base terminal groups can bind with uncoordinated Pb^2+^ ions to alleviate defects. Amino acid molecules serve as SAMs anchored on the TiO_2_ surface; these amino acid groups interact with the MA structure, mitigating defects caused by A-site vacancies in the perovskite [39]. This suggests that introducing L-EGT between the TiO_2_ layer and the perovskite helps to lower defects. The trap density (Nt) values for TiO_2_/MAPbI_3_ and TiO_2_/L-EGT/MAPbI_3_ are 5.53 × 10^15^ cm^−3^ and 2.43 × 10^15^ cm^−3^, respectively. The VTFL and Nt values for both structures are listed in Table S3. To further elucidate the reason behind the observed reduction in electrical properties (V_oc_, FF, and PCE) with prolonged L-EGT soaking duration, it was observed that after a soaking duration of 24 h, the trap-filled limit voltage (V_TFL_) rose from 0.11 V to 0.28 V, while the defect density escalated from 2.43 × 10^15^ cm^−3^ to 6.19 × 10^15^ cm^−3^, as depicted in Figure S3. These results elucidate that prolonged soaking duration in L-EGT leads to the formation of a large number of defects at the interface between the perovskite layer and the electron transport layer, which in turn create non-radiative recombination centers and adversely affect the electrical performance [40]. Additionally, from the analysis in Figure S4, the leakage current of the L-EGT-treated perovskite devices measured in the dark revealed that the dark current density near zero bias was 2.98 × 10^−9^ mA/cm^2^, lower than the control perovskite device (4.05 × 10^−9^ mA/cm^2^). However, when the L-EGT soaking time was extended to 24 h, the dark current density increased to 1.97 × 10^8^ mA/cm^2^, consistent with the I–V characteristics under dark conditions, indicating that the increased defect states contributed to the rise in leakage current. The reduction in dark current density further illustrates the passivation effect introduced by L-EGT. As shown in Figure 5b, the photoluminescence (PL) peak intensity significantly quenched after the introduction of L-EGT. The observed reduction in PL intensity suggests an improved ability to extract electrons, which can be attributed to the introduction of L-EGT positioned between the electron transport layer and the perovskite film. This enhancement in the efficiency of electron extraction signifies that the presence of L-EGT plays a crucial role in facilitating charge movement within the device, ultimately contributing to its overall performance.

To investigate the effects of incorporating L-EGT at the interface between the TiO_2_ and the perovskiteon carrier lifetime in perovskite devices, time-resolved photoluminescence (TRPL) measurements are conducted. The TRPL are analyzed utilizing a bi-exponential decay model, which allows us to determine the photoluminescence (PL) lifetimes through the following equation: *I(t) = A*_1_ *exp(–t/τ*_1_*) + A_2_ exp(–t/τ*_2_*) + I_0_*. In this equation, *A*_1_ and *A*_2_ denote the amplitudes of the decay, while τ represents the time constants associated with the decay. The fast decay lifetime (*τ*_1_) reflects non-radiative recombination processes that occur due to the trapping of carriers at the surface of the perovskite, while the slow decay lifetime (*τ*_2_) is indicative of radiative recombination occurring within the bulk of the perovskite [41,42,43]. The rapid quenching of the fast decay lifetime suggests that non-radiative recombination was effectively suppressed by passivating defects at the perovskite interface. Meanwhile, the quenching of the slow decay lifetime reflects an increase in charge transfer distance after charge separation. The analysis was performed on the ETL/perovskite solar cells with the following configurations: (a) original structure (control) and (b) L-EGT SAM-modified structure. Both τ_1_ and τ_2_ were reduced in the structure with L-EGT compared to TiO_2_/MAPbI_3_. The significant reduction in fast decay lifetime demonstrates that the introduction of L-EGT at the ETL/perovskite interface successfully passivated defects and suppressed non-radiative recombination. This enhancement in electron extraction capability is confirmed by the TRPL spectra shown in Figure 5c. In the absence of L-EGT, a longer PL lifetime is observed. However, with L-EGT incorporation, the τ_avg_ (average lifetime) significantly reduces to 24.4 ns from 56.4 ns, indicating that carriers are extracted more efficiently to the TiO_2_ layer, thereby reducing radiative recombination [44]. The pronounced quenching in TRPL lifetimes can be linked to the introduction of L-EGT, suggesting an enhancement in carrier transport dynamics at the interface [45,46]. The outcome of the calculated carrier lifetime of perovskite thin film is summarized in Table S4.

**Figure 6:**
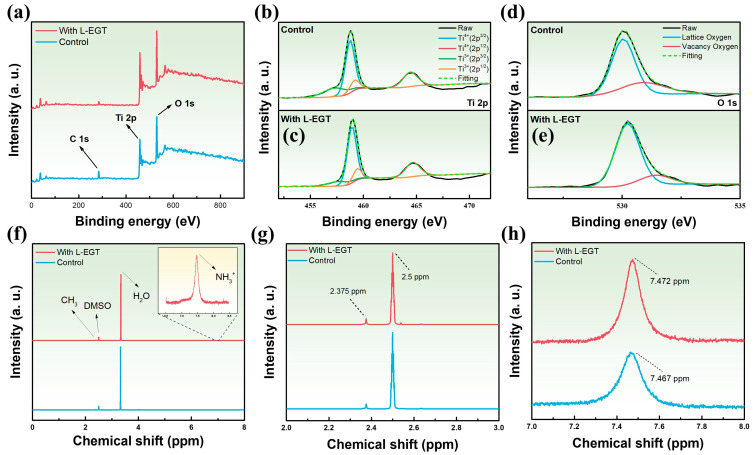
(**a**) XPS spectra of transport layer/perovskite interface with and without L-Ergothioneine modification. Fitting curves of Ti 2p (**b**) without L-Ergothioneine (Control), (**c**) with L-Ergothioneine. Fitting curves of O 1s (**d**) without L-Ergothioneine (Control), (**e**) with L-Ergothioneine. (**f**–**h**) Nuclear magnetic resonance (^1^H NMR) analysis of transport layer/perovskite interface with and without L-Ergothioneine modification.

We further investigated the interfacial reactions occurring in TiO_2_/L-EGT films utilizing XPS. The XPS spectra for Ti 2p and O 1s of TiO_2_ and TiO_2_/L-EGT are shown in Figure 6a. The spectra underwent calibration against the C 1s peak, which is established at 284.4 eV. Figure 6b,c present the XPS spectra of Ti 2p orbit for TiO_2_ and TiO_2_/L-EGT, respectively. By examining the variation in peak integration areas, we could observe stoichiometric changes as well as chemical bonding reactions between TiO_2_ and the ergothioneine group. In the sample with L-EGT, the fitted peaks at 458.91 eV, 464.64 eV, 457.65 eV, and 459.41 eV correspond to Ti^4+^ (2p^3/2^), Ti^4+^ (2p^1/2^), Ti^3+^ (2p^3/2^), and Ti^3+^ (2p^1/2^), respectively. Following the incorporation of L-EGT, the total peak area corresponding to Ti^4+^ rose from 70.75% to 79.15%. In contrast, the peak area associated with Ti^3+^ showed a decline, dropping from 29.24% to 20.85%. This variation suggests a significant change in the oxidation state of titanium within the material. The coordination between L-EGT and Ti^4+^ ions plays a crucial role in stabilizing the Ti^4+^ state, effectively inhibiting its reduction to the lower-valence Ti^3+^ ions. Table S5 provides the peak areas for the four distinct peaks observed at 458.91 eV, 464.64 eV, 457.65 eV, and 459.41 eV. In addition to the peak area changes, slight shifts in binding energy were observed, indicating the electronic state changes of Ti due to L-EGT incorporation [47]. Further analysis of the O 1s orbit for TiO_2_ and TiO_2_/L-EGT structure is shown in Figure 6d,e. The separate peaks observed at 530.23 eV and 531.44 eV are associated with lattice oxygen (OI) and oxygen vacancies (OII), respectively, and the fitting data of O 1s XPS are presented in Table S6. It is evident from the figures that the peak area of oxygen vacancies declined significantly after L-EGT was added, reducing from 29.21% to 15.81%. Moreover, the ratio of oxygen vacancies relative to lattice oxygen, calculated from the fitted O 1s spectra, decreased significantly from 0.412 to 0.187, reflecting a marked recline in lattice oxygen vacancies. Additionally, the O 1s spectra of the TiO_2_/L-EGT film showed an increase of about 0.2 eV in the binding energy of the OI peak, suggesting the formation of chemical bonds between TiO_2_ and the L-EGT group. The carboxyl (−COOH) group effectively filled the defects in the TiO_2_ electron transport layer and suppressed oxygen vacancies at the interface. Compared with previous studies [15], this research found that, in addition to forming bonds with the perovskite and suppressing PbI_2_ formation, L-EGT also interacts with TiO_2_. XPS analysis indicated that the introduction of L-EGT mitigated the reduction of Ti^4+^ to Ti^3+^, effectively passivating trap states within the TiO_2_ electron transport layer. To further analyze the reasons behind the increased defect density after prolonged L-EGT soaking, the XPS spectrum of the L-EGT SAM-modified film soaked for 24 h is shown in Figure S5. The Ti 2p orbit reveals that the peak area for Ti^4+^ declined from 79.15% to 70.41%, while the Ti^3+^ peak area increased from 20.85% to 29.59%, indicating partial reduction of Ti^4+^ to Ti^3+^ ions. Atomic hydrogen can reduce Ti^4+^ to Ti^3+^ [48,49]; an excess of atomic hydrogen dissociates into protons and electrons that bind to lattice oxygen, with the electrons being trapped at Ti sites, forming shallow energy levels [50,51].

Figure 6f illustrates the results of proton nuclear magnetic resonance (^1^H NMR) analysis, which was employed to examine the charge transport properties. The interaction between the amino groups of L-EGT and perovskite halides was examined through ^1^H NMR measurements, with signals obtained from the perovskite precursor solution. Liquid-state NMR was conducted before and after the addition of L-EGT to the perovskite solution. Further analysis of the liquid-state spectra of the perovskite precursor, both with and without L-EGT, revealed three characteristic peaks at δ = 2.375, δ = 2.502, and δ = 3.321 ppm. The peak at δ = 3.321 ppm stands for water, as DMSO has a strong water absorption capacity, resulting in a more prominent water signal in the spectrum [52]. The signal detected at around δ = 2.502 ppm is identified as coming from the solvent DMSO. In Figure 6g, a closer examination of the spectral region between 2 and 3 ppm uncovers that the peak observed at δ = 2.374 ppm is associated with the methylammonium (CH_3_) group. Additionally, in Figure 6h, a resonance signal around δ = 7.4648 ppm corresponds to NH_3_⁺ [53]. Upon mixing the solution with L-EGT, the characteristic peak of the methylammonium CH_3_ group (δ = 2.373 ppm) shows no significant shift. Notably, the interaction between the carboxyl group of L-EGT and the NH_3_⁺ group in methylammonium iodide (MAI) results in a defect passivation effect. A shift of 0.005 ppm in the NH_3_⁺ peak was observed, indicating a stronger interaction between NH_3_⁺ and L-EGT [54].

## 4. Conclusions

In this research, L-EGT was applied at the interface, aiming to enhance the interaction between the electron transport layer (TiO_2_) and the perovskite layer. SEM analysis demonstrated that the incorporation of L-EGT promoted the growth of perovskite grain size and led to a denser surface morphology. PL and TRPL indicated an enhancement in the efficiency of carrier extraction within the transport layer. The average lifetime dropped from 20.18 ns to 16.42 ns, indicating faster extraction of carriers to the TiO_2_ and diminishing the radiative recombination. It is hypothesized that the functional groups in L-EGT formed chemical bonds and suppressed oxygen vacancies at the interface, mitigating defect states and enhancing carrier transport performance.

XPS analyses indicated that the incorporation of L-EGT effectively mitigated oxygen vacancies within the lattice structure, while the increased concentration of Ti^4+^ ions contributed to enhanced passivation effects. In device applications, the introduction of L-EGT led to a notable improvement in the FF, rising from 75% to 78%. This enhancement is primarily attributed to a decrease in interfacial defects, better film quality, and overall improved device performance. The PCE also saw an increase from 16.88% to 17.84%. Remarkably, after 720 h, the performance still maintained approximately 91% of PCE. This stability suggests that the hydrogen atoms from the amino group (–NH_2_) of L-EGT played a crucial role in preventing the reduction of Ti^4+^ to Ti^3+^ ions, while the carboxyl group (–COOH) effectively filled the oxygen vacancies in TiO_2_, thereby passivating the defect states at the interface between the perovskite and the electron transport layer. Following the introduction of L-EGT, the Ti^4+^ content increased from 70.75% to 79.15%, while oxygen vacancies were dropped from 29.21% to 15.81%. Additionally, the –NH_2_ group of L-EGT can establish hydrogen bonds with methylammonium (MA) ions, further bolstering the stability and longevity of the perovskite film. The results indicate that the incorporation of L-EGT into the perovskite structure enhances and facilitates the formation of bonds with the perovskite layer while establishing an anchoring effect with TiO_2_, thereby potentially extending the operational lifetime of perovskite solar cells.

## Data Availability

Data are contained within the article or Supplementary Materials.

## Supplementary Materials

The following supporting information can be downloaded at: https://www.mdpi.com/article/10.3390/ma17235739/s1, Figure S1. (a) Chemical structure of L-Ergothioneine (b) Schematic of perovskite solar cell with L-Ergothioneine; Figure S2: The Current–Voltage (*I–V*) curve characteristic of the perovskite solar cell with the L-Ergothioneine and without L-Ergothioneine at different aging duration; Figure S3. Current density–voltage curves and trap density of perovskite films with L-Ergothioneine (Long soaking time) and without L-Ergothioneine (control) modification; Figure S4: Dark Current-Voltage (I–V) curves of the perovskite solar cell with the L-Ergothioneine and without L-Ergothioneine (control) modification; Figure S5: XPS spectra of (a) Ti 2p (b) O 1s electron transport layer/perovskite interface fitting curves with L-Ergothioneine for soaking 24 h; Table S1. Photovoltaic performance of perovskite solar cells with different deposition times and concentration of L-Ergothioneine; Table S2. Summary of different SAM Materials; Table S3. Trap-Filling Limited Voltage (V_TFL_) and trap density (N_t_) calculation of perovskite solar cell with only electron transport layer structure under varying soaking duration from SCLC measurement; Table S4. Calculated carrier lifetime of perovskite thin film deposited on TiO_2_ with and without L-Ergothioneine modification; Table S5. XPS fitting data of Ti 2p with and without L-Ergothioneine modification; Table S6. XPS fitting data of O 1s with and without L-Ergothioneine modification.
