# Supplementary material for: Suppressing Interface Defects in Perovskite Solar Cells via Introducing a Plant-Derived Ergothioneine Self-Assembled Monolayer
Source: Materials (Basel). 2024 Nov 23;17(23):5739. doi: 10.3390/ma17235739 (PMC11641942; doi:10.3390/ma17235739)
Supplement: Supplementary file 1 [file materials-17-05739-s001.zip › materials-3311840-supplementary.pdf]

Supplementary information

Suppressing Interface Defects in Perovskite Solar Cells via Introducing a Plant-Derived Ergothioneine Self-Assembled Monolayer

Cheng-Hsien Yeh [1,2,†], Hung-Chieh Hsu [1,3,†], Jung-Che Tsao [1,3], Hsuan-Ta Wu [4], Teh-Pei Lin [5], Chien-Te Wu [5], Shih-Hsiung Wu [3,*] and Chuan-Feng Shih [1,2,6,*]

[1] Department of Electrical Engineering, National Cheng Kung University, Tainan 70101, Taiwan
[2] Applied High Entropy Technology (AHET) Center, National Cheng Kung University, Tainan 70101, Taiwan
[3] Green Energy and Environment Research Laboratories, Industrial Technology Research Institute, Tainan 711010, Taiwan
[4] Department and Institute of Electrical Engineering, Minghsin University of Science and Technology, Hsinchu 30401, Taiwan
[5] Institute of Green Products, Feng Chia University, Taichung 40724, Taiwan
[6] Hierarchical Green-Energy Materials (Hi-GEM) Research Center, National Cheng Kung University, Tainan 70101, Taiwan
* Correspondence: shihhsiung@itri.org.tw (S.-H.W.); cfshih@mail.ncku.edu.tw (C.-F.S.)
† These authors contributed equally to this work.

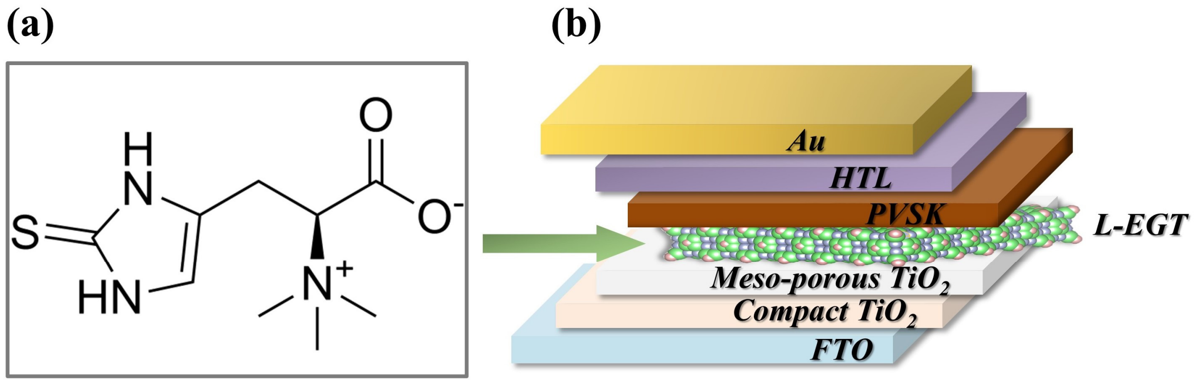

Figure S1. (a) Chemical structure of L-Ergothioneine (b) Schematic of perovskite solar cell with L-Ergothioneine

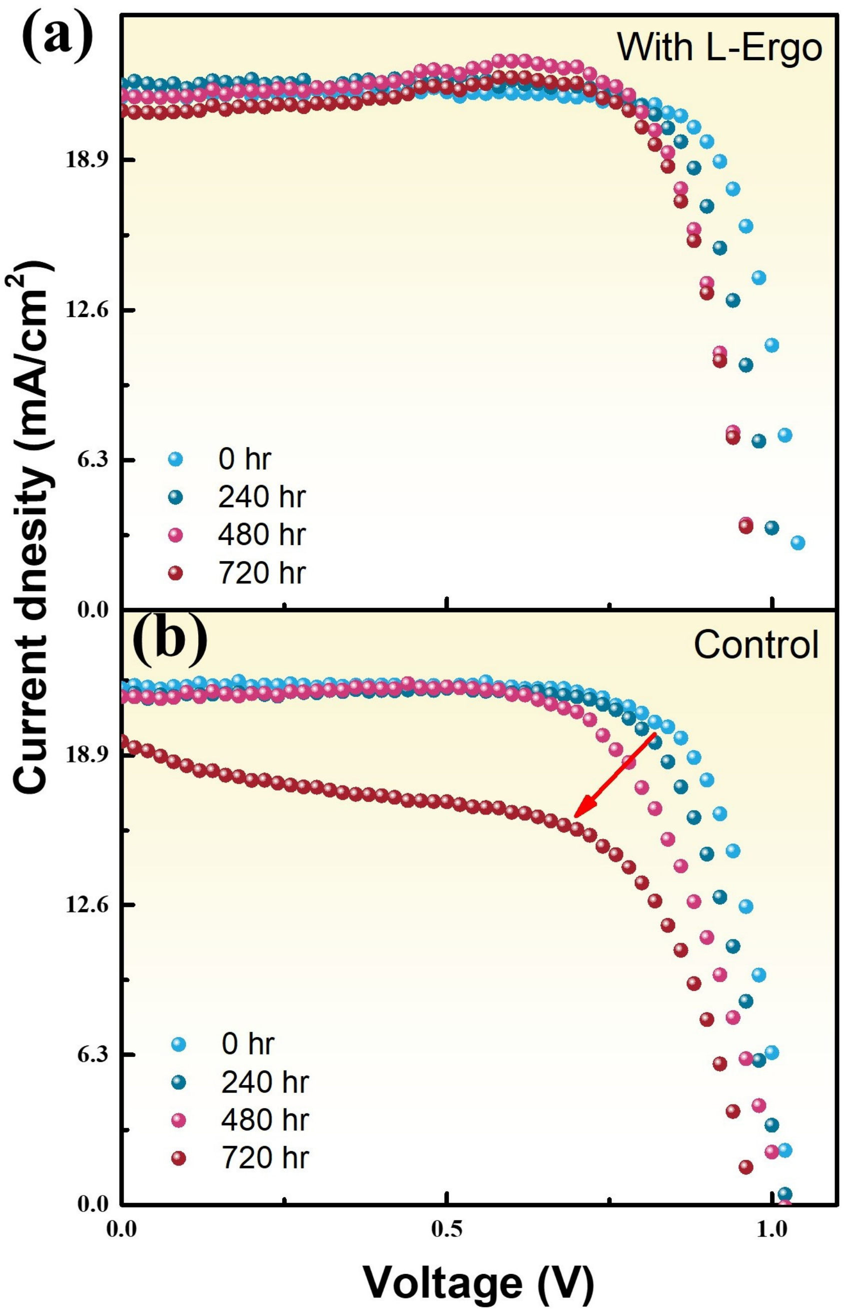

Figure S2. The Current-Voltage (*I–V*) curve characteristic of the perovskite solar cell with the L-Ergothioneine and without L-Ergothioneine at different aging duration.

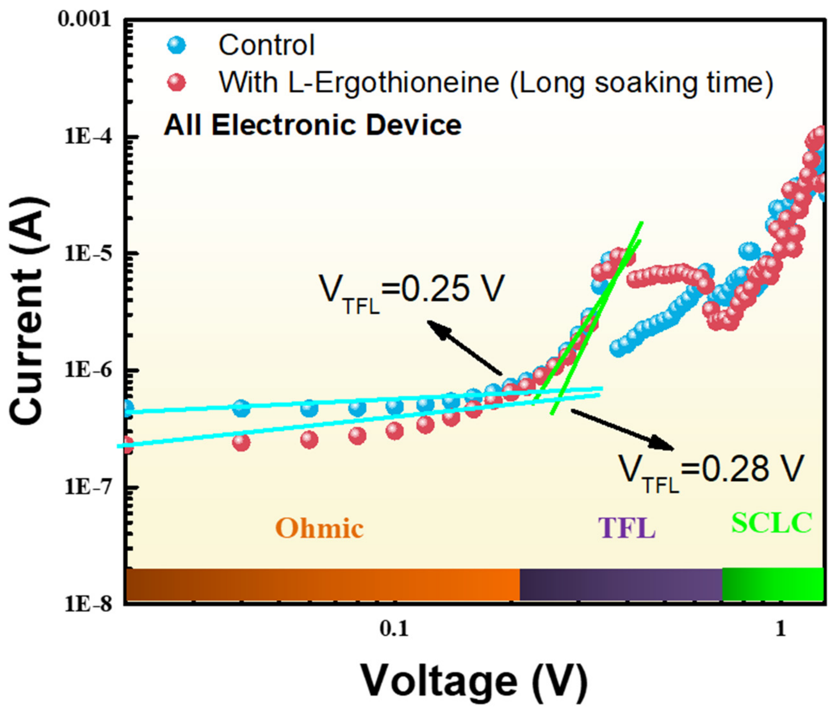

Figure S3. Current density–voltage curves and trap density of perovskite films with L-Ergothioneine (Long soaking time) and without L-Ergothioneine (control) modification.

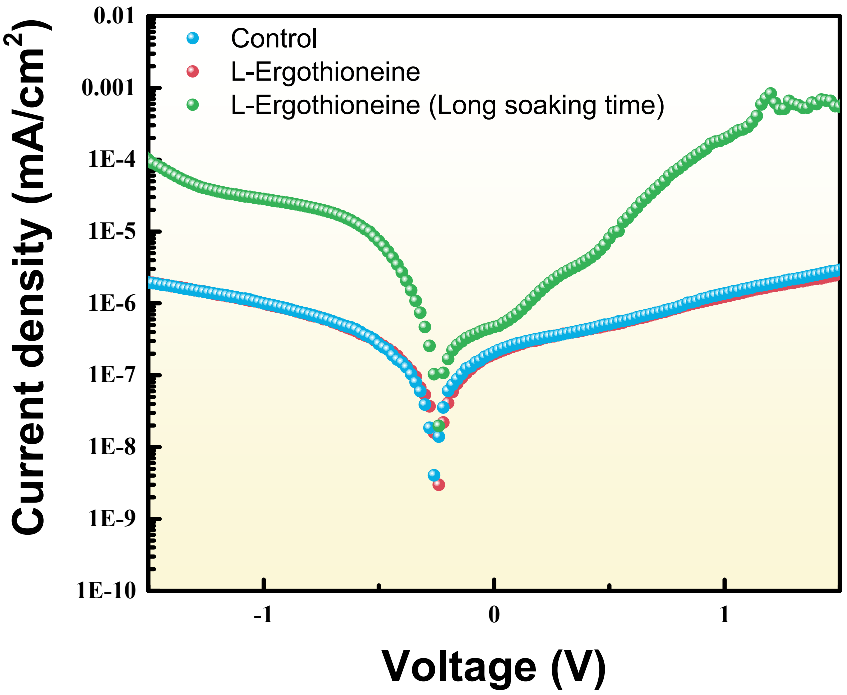

Figure S4. Dark Current-Voltage (*I–V*) curves of the perovskite solar cell with the L-Ergothioneine and without L-Ergothioneine (control) modification.

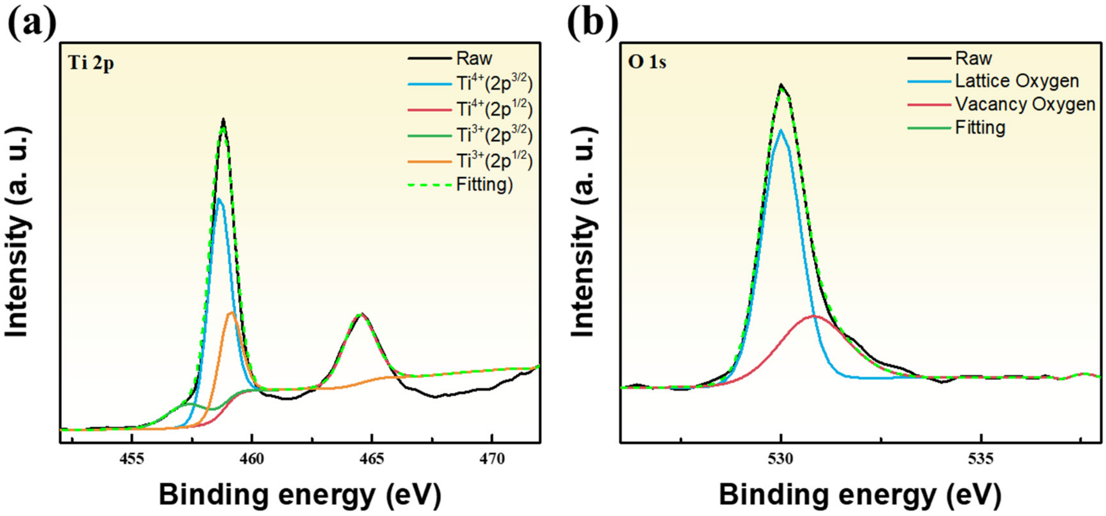

Figure S5. XPS spectra of (a) Ti 2p (b) O 1s electron transport layer/perovskite interface fitting curves with L-Ergothioneine for soaking 24 hours.

Table S1. Photovoltaic performance of perovskite solar cells with different deposition times and concentration of L-Ergothioneine.

Structure	*Jsc* (mA/cm^2)	*Voc* (V)	*FF* (%)	*PCE* (%)
**Control**	21.71	1.02	75	16.88
**0.1mM (60 min)**	21.98	1.02	76	17.30
**0.3mM (5 min)**	21.75	1.03	77	17.48
**0.3mM (60 min)**	21.53	1.05	78	17.84
**0.6mM (5 min)**	21.60	1.02	77	17.12
**0.6mM (60 min)**	21.80	1.00	75	16.40

Table S2. Summary of different SAM Materials

SAM material	Aging time	Remaining of original PCE	Reference
**glycine**	**840 hours**	**78 %**	**[5]**
**L-Ergothione**	**720 hours (T of 85 °C and RH of 20%)**	**67 %**	**[14]**
**HO2C–PP–NH2 (PP = biphenyl)**	**148 hours**	**67 %**	**[49]**
**dopamine (DA)**	**300 hours**	**80%**	**[27]**
**L-Histidine**	**500 hours**	**91 %**	**[23]**
**L-Ergothione**	**720 hours**	**91%**	**This work**

Table S3. Trap-Filling Limited Voltage (V_{TFL}) and trap density (N_t) calculation of perovskite solar cell with only electron transport layer structure under varying soaking duration from SCLC measurement.

**Structure**	V_{TFL} **(V)**	N_t **(cm^{-3})**
**Control**	0.25	$5.53x10^{15}$
**With L-Ergo**	0.11	$2.43x10^{15}$
**With L-Ergo (24 hours)**	0.28	$6.19x10^{15}$

Table S4. Fitting parameters of TRPL spectra for perovskite thin-film deposited on TiO_2 with and without L-Ergothioneine modification.

**Structure**	τ_{avg} ***(ns)***	τ_1 ***(ns)***	τ_2 ***(ns)***
**Control**	20.18	25.68	10.99
**With L-Ergo**	16.42	20.16	8.11

Table S5. XPS fitting data of Ti 2p with and without L-Ergothioneine modification.

	Ti^{4+} ($2p^{3/2}$)	Ti^{4+} ($2p^{1/2}$)	Ti^{3+} ($2p^{3/2}$)	Ti^{3+} ($2p^{1/2}$)	Ti^{4+}	Ti^{3+}
**Control**	49.35 % (458.74 eV)	21.4 % (464.41 eV)	19.77 % (457.29 eV)	9.47 % (459.16 eV)	70.75 %	29.24 %
**With L-Ergo**	56.53 % (458.91 eV)	22.62 % (464.64 eV)	10.5 % (457.65 eV)	10.35 % (459.41eV)	79.15 %	20.85 %
**With L-Ergo (24 hours)**	48.46 % (458.64 eV)	21.95% (464.47 eV)	9.52 % (457.3 eV)	20.07 % (459.07 eV)	70.41 %	29.59 %

Table S6. XPS fitting data of O 1s with and without L-Ergothioneine modification.

	**Lattice oxygen**	**Oxygen vacancy**
**Control**	70.79 %<br>(530.04 eV)	29.21 %<br>(530.83 eV)
**With L-Ergo**	84.19 %<br>(530.23 eV)	15.81 %<br>(531.44eV)
**With L-Ergo (24 hours)**	69 %<br>(530.01 eV)	31 %<br>(530.79 eV)
